# In Vitro Evaluation of a Solid Supersaturated Self Nanoemulsifying Drug Delivery System (Super-SNEDDS) of Aprepitant for Enhanced Solubility

**DOI:** 10.3390/ph14111089

**Published:** 2021-10-27

**Authors:** Hakan Nazlı, Burcu Mesut, Yıldız Özsoy

**Affiliations:** 1 Department of Pharmaceutical Technology, Faculty of Pharmacy, Trakya University, Edirne 22030, Turkey; hakannazli@trakya.edu.tr; 2 Department of Pharmaceutical Technology, Faculty of Pharmacy, Istanbul University, Istanbul 34116, Turkey; bmesut@istanbul.edu.tr

**Keywords:** aprepitant, supersaturated SNEDDS, solid SNEDDS, solubility enhancement, soluplus^®^, imwitor^®^ 988

## Abstract

Aprepitant (APR) belongs to Class II of the Biopharmaceutical Classification System (BCS) because of its low aqueous solubility. The objective of the current work is to develop self-nanoemulsifying drug delivery systems (SNEDDS) of APR to enhance its aqueous solubility. Preformulation studies involving screening of excipients for solubility and emulsification efficiency were carried out. Pseudo ternary phase diagrams were constructed with blends of oil (Imwitor^®^ 988), cosolvent (Transcutol^®^ P), and various surfactants (Kolliphor^®^ RH40, Kolliphor^®^ ELP, Kolliphor^®^ HS15). The prepared SNEDDS were characterized for droplet size and nanoemulsion stability after dilution. Supersaturated SNEDDS (super-SNEDDS) were prepared to increase the quantity of loaded APR into the formulations. HPMC, PVP, PVP/VA, and Soluplus^®^ were used as polymeric precipitation inhibitors (PPI). PPIs were added to the formulations at 5% and 10% by weight. The influence of the PPIs on drug precipitation was investigated. In vitro lipolysis test was carried out to simulate digestion of formulations in the gastrointestinal tract. Optimized super-SNEDDS were formulated into free-flowing granules by adsorption on the porous carriers such as Neusilin^®^ US2. In vitro dissolution studies of solid super-SNEDDS formulation revealed an increased dissolution rate of the drug due to enhanced solubility. Consequently, a formulation to improve the solubility and potentially bioavailability of the drug was developed.

## 1. Introduction

Aprepitant (APR) is the first commercially available drug from a new class of neurokinin-1 (NK1) receptor antagonists. It acts by reducing the emetic effects of substance P [1]. Substance P is a neuropeptide consisting of 11 amino acids and is an endogenous ligand for NK1 receptors. It acts as a neurotransmitter and is involved in the development of inflammatory and immune responses in the body [2]. Substance P is the most abundant neurokinin in the central nervous system of mammals and plays a key role in the pathophysiology of many events such as delayed emesis due to chemotherapy [1]. APR is used orally in combination with other agents (like corticosteroids) for the prevention of acute and delayed nausea and vomiting associated with highly and moderately emetogenic chemotherapy and for the prevention of post-operative nausea and vomiting [3].

**Figure 1:**
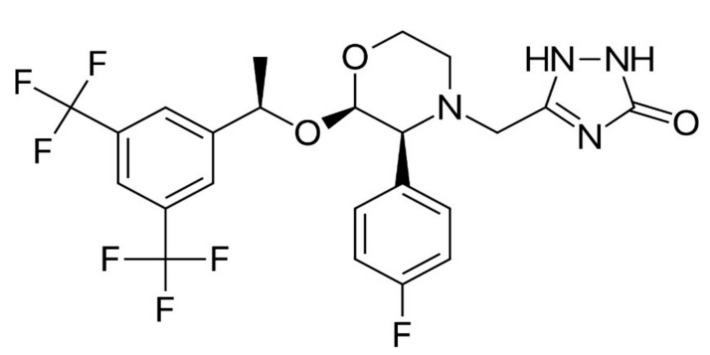
Chemical structure of APR.

In appearance, APR is a white to off-white, non-hygroscopic, crystalline solid powder. The molecular weight of APR is 534.4 g/mol and its chemical structure is 5-(((2*R*,3*S*)-2-((1*R*)-1-(3,5- bis(trifluoromethyl)phenyl)ethoxy)-3-(4-fluorophenyl)4-morpholinyl)methyl)-1,2-dihydro-3*H*-1,2,4-triazol-3-one [4]. The structure of APR is given in Figure 1.

APR is a weakly basic compound with a pKa of 9.7. It is a relatively highly lipophilic compound since the logP value is 4.8 at neutral pH. It melts at 254 °C [5]. It is practically insoluble in water, sparingly soluble in ethanol and isopropyl acetate, slightly soluble in acetonitrile [6]. According to different literature data, APR is categorized as Biopharmaceutical Classification System (BCS) Class II [7] or Class IV [8] molecule. The moderate permeability of APR is the reason for this.

Water insolubility is one of the main elements which restrict the usage of many potential drug compounds [9]. Insoluble drugs in the gastrointestinal tract cause slow drug release rates and generally exhibit erratic and incomplete absorption. Because of these reasons the bioavailability of APR is inadequate [10]. Due to the disadvantages of poor solubility, it is not possible to provide an effective treatment using conventional formulations of APR. In order to increase the solubility, various salt forms of APR were prepared before commercialization. However, it was observed chemical stability of prepared salts was weak. Thus, a particle size reduction method is used in the production of commercialized drug (Emend^®^) [11].

NanoCrystal^®^ technology (Elan Corporation, Dublin/Ireland) is used in the Emend^®^, in which its particles are ground to submicron size and stabilized with polymer or surfactant [12]. Excipients used in Emend^®^ are hydroxypropyl cellulose, sodium lauryl sulfate, and sucrose. During the particle size reduction, hydroxypropyl cellulose acts as a stabilizer. Sodium lauryl sulfate is used to increase the efficiency of the grinding process by reducing the media viscosity. In addition, it also acts as a lubricant in capsule filling. Sucrose ensures that the nanoparticles in colloidal suspension remain stable during the drying process [13].

APR displays nonlinear pharmacokinetics. Bioavailability of APR decreases with increasing dose. For example, the mean oral bioavailability of APR is 67% for the 80 mg Emend^®^ capsule and 59% for the 125 mg Emend^®^ capsule. The mean peak plasma concentration of APR occurred at approximately 4 h [14]. With the aid of nano-sized formulation of APR, nonlinear pharmacokinetics in fasted conditions could be overcome and the food effect was eliminated. As a result, a more suitable dosage form in terms of bioavailability was produced [15]. However, particle size reduction methods have some disadvantages such as high production costs. Therefore, attempts to increase the solubility and dissolution rate of APR with other techniques have been continued [11].

There are different methods for increasing the solubility and dissolution rate of the APR, such as solid dispersions, cyclodextrin complexes, nanoparticles, lipid-based drug delivery systems [16]. Self-nanoemulsifying drug systems (SNEDDS) are isotropic mixtures that spontaneously form *o*/*w* type nanoemulsions with droplet sizes usually below 200 nm when mixed with an aqueous phase under mild agitation. SNEDDS can contain excipients such as oils, surfactants, cosurfactants, and solubilizers in order to facilitate nanoemulsification or improve the solubility of the drug in the formulation [17]. SNEDDS have some advantages when compared to emulsions or nanoemulsions. For example, SNEDDS formulations are physically more stable, their scale-up and manufacturing in large-scale processes are easier, they can be converted into unit dosage forms which improve patient compliance [17,18].

The present study is aimed to design and develop APR-loaded SNEDDS with the objective of increasing its solubility and bioavailability. Firstly, suitable excipients were selected from the large pool of excipients. Then, the appropriate ratios of these excipients are determined. In vitro characterizations of formulations that have suitable properties were performed.

## 2. Results and Discussion

### 2.1. Method for Quantification of APR

The standard curve used to determine the concentration of APR showed good linearity over the concentration range of 1–20 µg/mL. The r^2^ value for the standard curve was above 0.999. Intra-day and inter-day variations of RSD were below ≤2%, and mean recovery was between 98% and 102%. The limit of detection (LOD) and limit of quantification (LOQ) values were 0.3 and 0.8 µg/mL, respectively. The retention time of APR was 7.3 ± 0.1 min.

### 2.2. Equilibrium Solubility of APR

The solubility data obtained is not the exact solubility values of the aprepitant in the relevant mediums, but represent the apparent solubility values after 24 h. Apparent solubility is the experimentally determined solubility of a solute in a solvent system and may be higher or lower than the equilibrium solubility (equilibrium solubility) due to temporary supersaturation, incomplete dissolution, or insufficient time [19]. At this stage, exact thermodynamic solubility values are not necessary, and the apparent solubility approach is sufficient to eliminate the available excipients [20]. The key aspect to developing a successful SNEDDS formulation is the selection of suitable excipients. Drug solubilization and nanoemulsification performance of excipients should be taken into consideration. Considering the clinically used doses of APR drug loading capacity of SNEDDS formulation is vital. Choice of oil and cosolvent was based on the drug solubilization.

**Figure 2:**
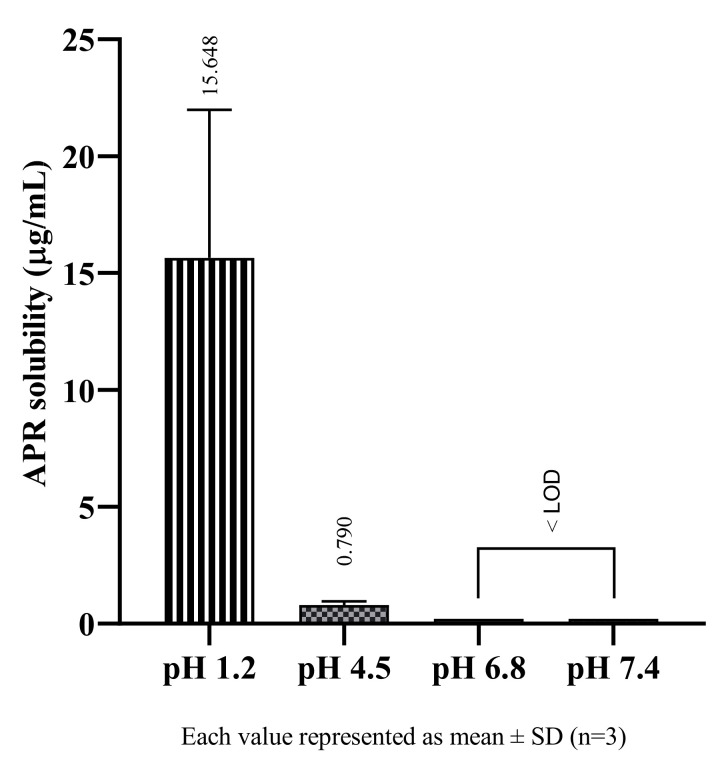
The solubility of APR at 37 °C in buffer solutions.

Studies conducted in buffer solutions to determine the solubility of APR in physiological environments, it was observed that APR showed pH-dependent solubility and had higher solubility in acidic environments. Since APR is a weakly basic drug, it is an expected result. Weakly basic drugs are ionized in acidic mediums, so their solubility increases [21]. The solubility of APR in buffers are shown in Figure 2. The physiological pH in which APR has the best solubility is pH 1.2 at 37 °C. However, the result shows that APR is practically insoluble even at low pH. As the pH value increased, the solubility of APR decreased, and it could not be determined as it fell below LOD in pH 6.8 and pH 7.4.

**Figure 3:**
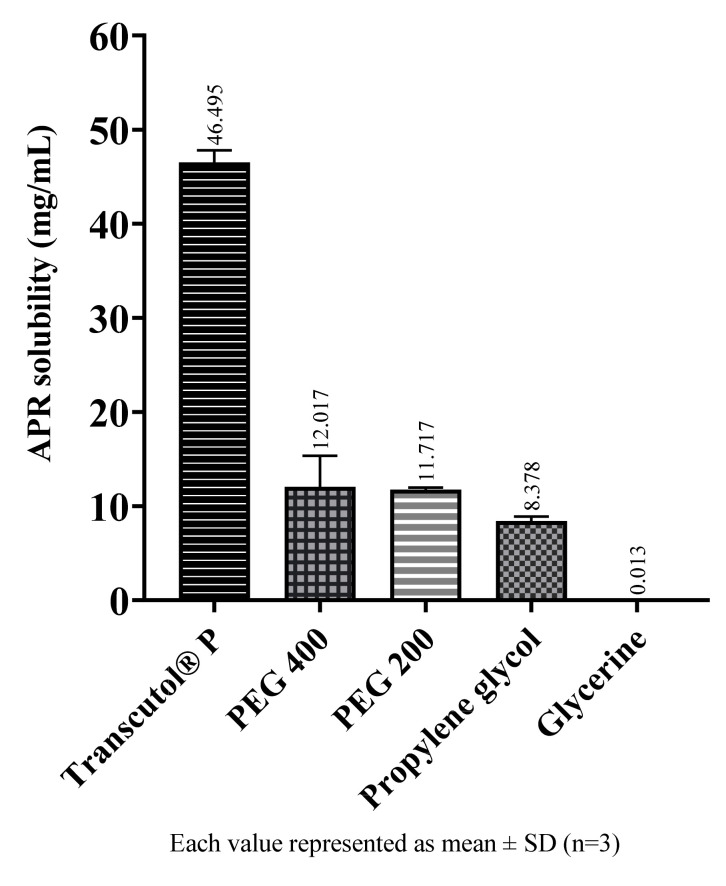
The solubility of APR at 25 °C in cosolvents.

The solubility of APR in cosolvents are shown in Figure 3. Among the cosolvents, Transcutol^®^ P dissolves a much more amount of APR than the others. Transcutol^®^ P is a known excipient and is frequently used as a cosolvent since it is a capable solubilizer for many drugs with low water solubility.

**Figure 4:**
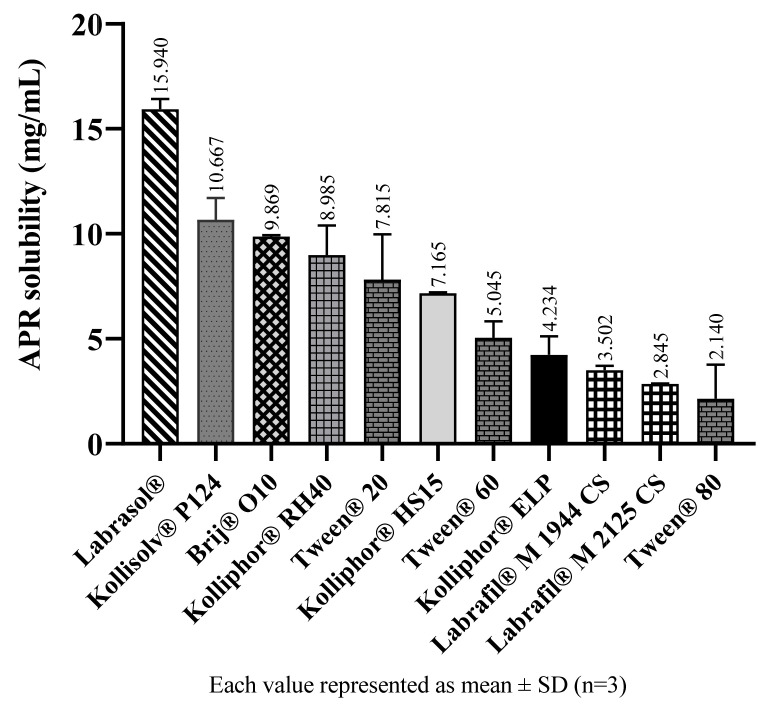
The solubility of APR at 25 °C in surfactants.

**Figure 5:**
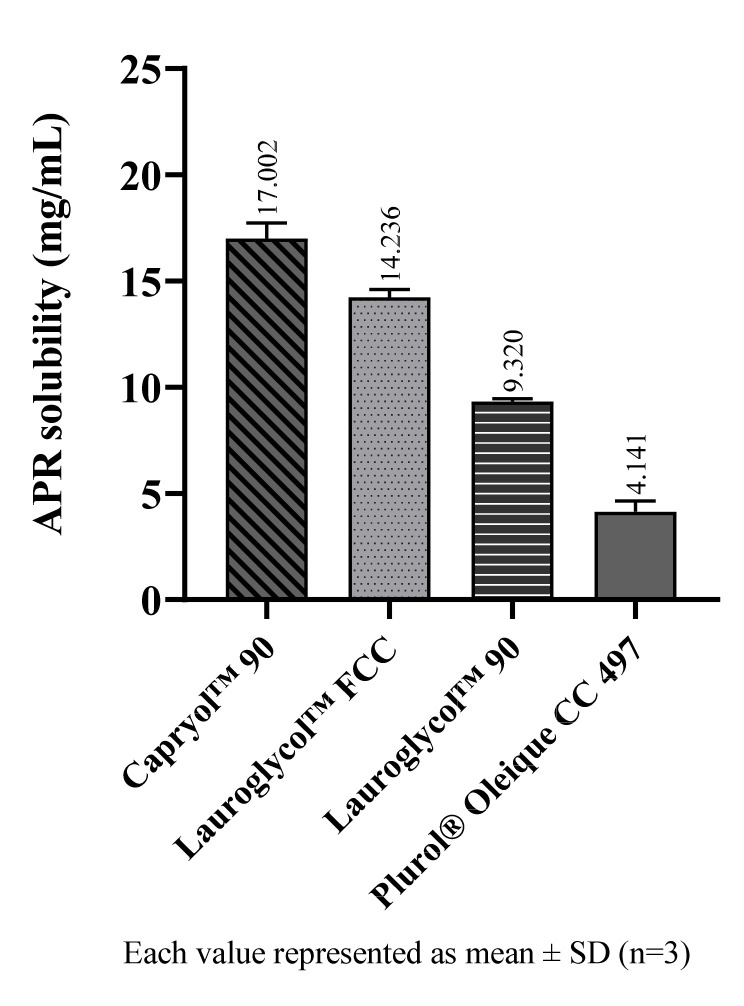
The solubility of APR at 25 °C in cosurfactants.

In SNEDDS formulations, emulsification efficiency plays a more vital role than solubility while choosing surfactants and cosurfactants [22]. However, solubility data can help to determine which surfactants should be preferred with similar emulsification efficiency. Results from solubility studies in surfactants suggest the amount of APR that can be loaded into formulations. The solubility of APR in surfactants and cosolvents are shown in Figure 4 and Figure 5, respectively.

**Figure 6:**
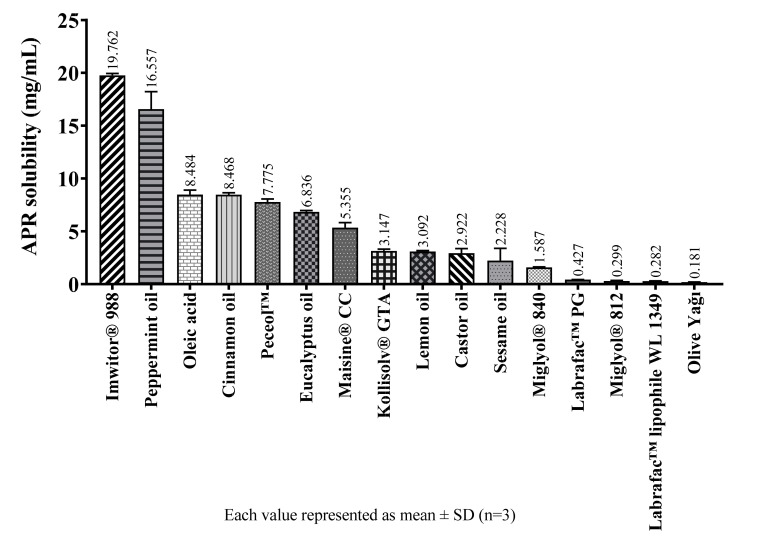
The solubility of APR at 25 °C in oily excipients.

The solubility of APR in various oily excipients are shown in Figure 6.

Solubility studies performed with oily substances show that medium-chain partial glycerides dissolve more APR than corresponding long-chain unsaturated derivatives. The solubility of APR decrease as the chain length of glycerides increase. The same is true for propylene glycol esters. The solubility of APR is low in oily substances which do not have surface-active properties such as medium-chain triglycerides (MCTs) or long-chain triglycerides (LCTs). Since APR is a brick dust-like molecule, its low solubility in MCTs is an expected result. The oily substance with the highest APR solubility is Imwitor^®^ 988. Imwitor^®^ 988 is a glycerol ester of caprylic (C_8_) and capric (C_10_) acid. Most of this mixture (>90%) consists of caprylic acid. The ester part mainly contains monoglyceride (45–75%) and diglyceride (20–50%) fractions. Triglyceride fraction is less than 10% [23]. Since monoglyceride and diglyceride fractions are not completely esterified, they have surface-active properties and differ from triglycerides in terms of solubility properties [24]. It is considered that the reason for the relatively high solubility of APR in Imwitor^®^ 988 is due to having the monoglyceride and diglyceride structures which have surface-active properties.

Alskär et al. [25] investigated the solubility of 35 water-insoluble drugs in excipients used in lipid-based formulations. They found that the order of drug solubility was cosolvents > surfactants > medium-chain triglycerides > long-chain triglycerides. They also stated that mixtures of glycerides had higher solvent capacity than triglycerides. The findings of this work and our results are compatible with each other.

The tight crystal lattice might be the cause of why it is not readily soluble in oils, despite having a relatively high logP (4.8) value [26]. The high melting temperature (254 °C) also indicates the tightness of the crystal structure of the APR.

### 2.3. Screening of Surfactants

Although being a substantial step in choosing the ingredients of SNEDDS, drug solubility is not the only parameter. The emulsifying efficiency of the surfactant is much more important [22]. Therefore, surfactant screening was performed according to the selected oil. Imwitor^®^ 988 was chosen as the oil phase because dissolving more APR than other oily excipients. Screening of surfactant was conducted for Imwitor^®^ 988, and percentage transmittance values for various surfactants were measured. The HLB values of the most appropriate surfactants for Imwitor^®^ 988 were in the range of 14-16. The emulsification efficiency of surfactants with lower HLB values, such as Labrasol^®^ and Labrafil^®^ types were poor. High percentage transmittance values indicated that the oil droplets were in nano size [27]. The percentage transmittance values for tested surfactants are shown in Table 1. Kolliphor^®^ RH40 was chosen as a surfactant due to its better nanoemulsification efficiency.

### 2.4. Screening of Cosurfactants and Cosolvents

Suitable cosurfactants or cosolvents for the various oil and surfactant mixtures were evaluated for emulsification efficiency. The percentage transmittance values for tested cosurfactants and cosolvents are shown in Table 2.

In order to better understand how the added cosurfactants or cosolvents affect the turbidity, the results obtained in the surfactant selection findings are shown in the table as shaded rows. All excipients, except Lauroglycol™ FCC, have been shown to reduce turbidity and thus improve emulsification efficiency for Imwitor^®^ 988–Kolliphor^®^ RH40 mixture. Cosurfactants can improve the emulsification of surfactants by penetrating interfacial monolayer and play a role in further reducing interfacial tension [28].

Since the percentage transmittance values are very close to each other, Transcutol^®^ P, which can dissolve the highest amount of APR among all tested excipients, was chosen as a cosolvent. Thus, the loading capacity of SNEDDS preconcentrate can be increased.

### 2.5. Construction of Pseudo Ternary Phase Diagrams

**Figure 7:**
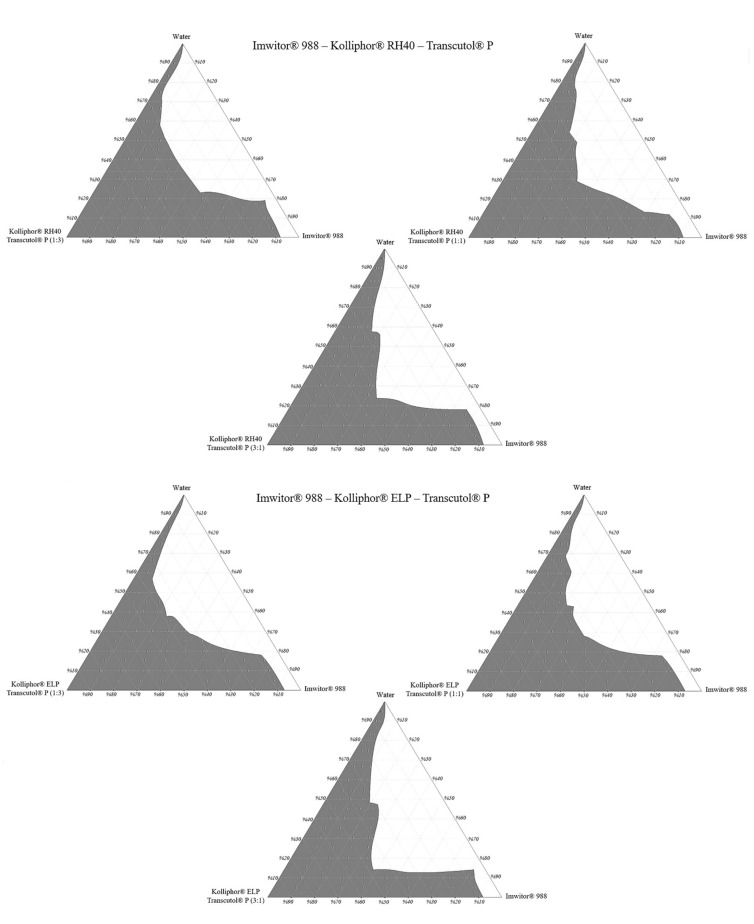

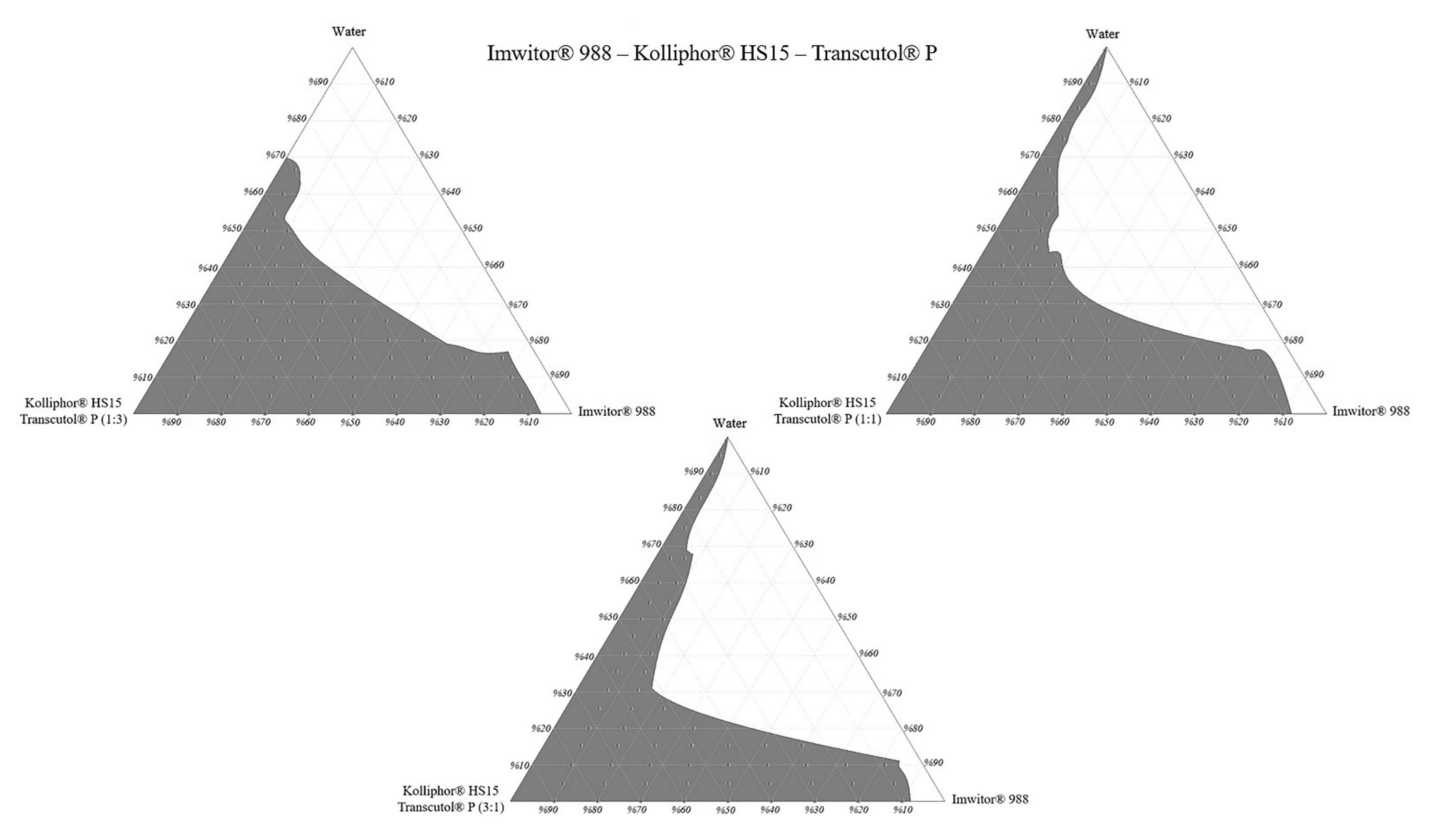
Pseudo ternary phase diagrams.

The construction of phase diagrams is a step as essential as the solubility tests [20]. Principally, the phase diagrams are used to explain different phases in equilibrium, such as in a thermodynamically stable microemulsion system. Since nanoemulsions are thermodynamically unstable, there is no actual equilibrium between the phases. Therefore, in this study, pseudo ternary phase diagrams were just constructed to determine the excipient ratios that can form a nanoemulsion [29]. The nanoemulsion areas of the pseudo ternary phase diagrams were compared. A larger area meant higher nanoemulsification efficiency. The nanoemulsion region was defined where clear dispersions were obtained when water was added dropwise to the formulation. Pseudo ternary phase diagrams are shown in Figure 7.

Pseudo ternary phase diagrams showed that the area of nanoemulsion (the gray shaded area) was highest in formulations prepared with Imwitor^®^ 988–Kolliphor^®^ RH40 –Transcutol^®^ P mixture at a 3:1 Smix ratio. For Imwitor^®^ 988, the highest total area was obtained with Kolliphor^®^ RH40. This surfactant can nanoemulsify up to 30% Imwitor^®^ 988 in formulations. Areas of the pseudo ternary phase diagrams are shown in Table 3.

The ternary phase diagram area is not only affected by the type of surfactant but also by the ratio of the cosolvent in Smix. The effect of the Smix ratio on the pseudo ternary phase diagram area may vary according to the excipients used in the formulation. In some cases, increasing the surfactant ratio in the Smix leads to an area increase. This could be due to increased surfactant amount will lower the surface tension, which leads to the formation of a more stable nanoemulsion [30]. However, in some other cases, it is also possible to observe the reverse of this situation. SNEDDS excipients can form gel-like or creamy viscous structures when used in specific proportions [31]. In this case, increasing the cosolvent amount may cause an increase in the area in the ternary phase diagrams.

As a result, Imwitor^®^ 988, Kolliphor^®^ RH40, Transcutol^®^ P was selected as oil, surfactant, and cosolvent, respectively. The formulations are coded as the oil:surfactant:cosolvent ratio.

### 2.6. Nanoemulsion Droplet Size Measurements in Blank Formulations

The droplet size of SNEDDS is a critical property for enhancing oral drug bioavailability. Dynamic light scattering (DLS) is generally preferred for routine measurements in measuring droplet size [32]. Although the reliability of DLS results in samples with complex structures is controversial, it is thought the reliability of DLS results in this work was high since the selected formulations showed unimodal distributions with low polydispersity. Z-average and low polydispersity index (PDI) values of the blank formulations prepared with different excipient ratios are shown in Table 4.

Formulations with a Z-average value of less than 150 nm and a PDI value of less than 0.25 with a unimodal distribution were considered suitable.

### 2.7. APR Loading Capacity of Formulations

**Figure 8:**
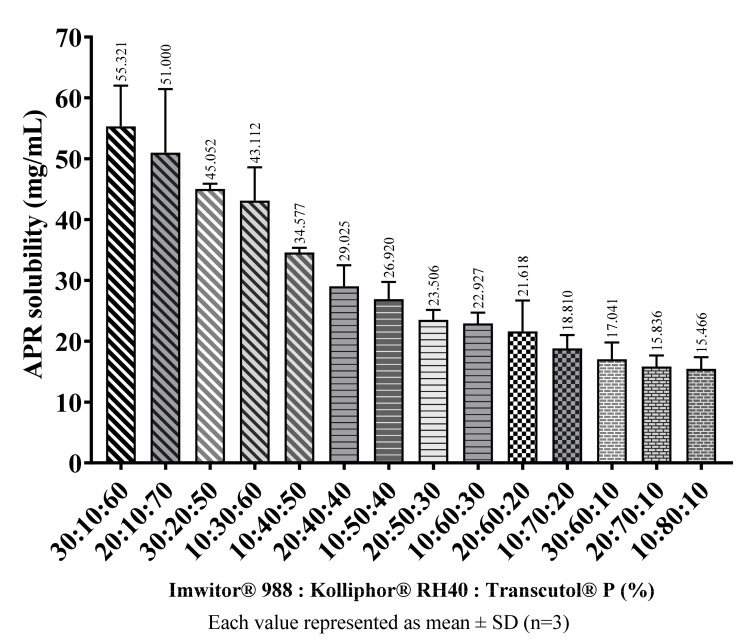
The loading capacity of SNEDDS preconcentrates at 37 °C.

APR amounts that can be loaded into blank formulations considered suitable were determined as described in the methods (Equilibrium solubility of APR) section. The main factor determining the solubility of APR in formulations was the ratio of cosolvents in the formulations. In general, as the amount of Transcutol^®^ P in the formulation increased, the amount of APR that the formulation can dissolve also increased. The amount of APR in saturated SNEDDS preconcentrates are shown in Figure 8.

### 2.8. Nanoemulsion Droplet Size Measurements in APR Loaded Formulations

It was aimed to load at least 21 mg APR in 1 g of SNEDDS formulation. Aprepitant was loaded only on the blank formulations that droplet size and PDI value were considered suitable. Measurements were repeated at 0, 1, 2, and 4 h, and changes in the droplet size were investigated to examine the stability of the formed nanoemulsions. The result of this study is presented in Table 5.

The 10:70:20, 30:60:10, 20:70:10, and 10:80:10 formulations could not be loaded with desired amounts of APR because the dissolving capacity of the preconcentrates was not sufficient. DLS measurements also could not be performed because rapid drug precipitation was observed in formulations (such as 30:10:60 and 20:10:70) loaded with 40 mg APR. Some formulations loaded with APR (such as 10:30:60) showed severe deterioration in droplet size and PDI over time as a result of dilution 100-fold with distilled water. Droplet size measurements indicated that adding APR to SNEDDS had a notable effect. Adding APR in formulations could influence nanoemulsification due to APR could interact with the oil-water interface [33]. The increase in droplet size and PDI might also due to the precipitation of APR as a result of the reduced solubilisation capacity caused by the dilution [34].

It was observed that the droplet size growth rate increased as the amount of loaded APR increased. For example, blank 10:30:60 formulation has a narrow droplet size (15.02 ± 2.43) distribution. The period that the droplet size remains stable decreases as the amount of added APR increases from 21 mg to 30 mg. That could be attributed to the supersaturated degree after dilution. Since the loaded APR increases, it gets harder to maintain the supersaturated state for an extended period [29].

### 2.9. In Vitro Drug Precipitation Test from Saturated SNEDDS

**Figure 9:**
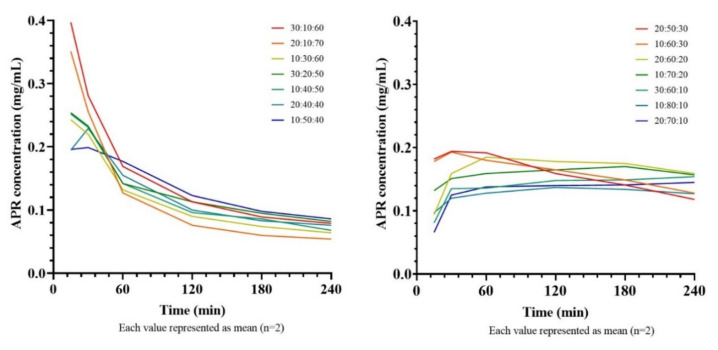
Concentration-time curves of saturated SNEDDS formulations.

Since the nanoemulsions formed after the dilution of SNEDDS are not thermodynamically stable, it is expected that the active substances will precipitate over time. It has been shown that the precipitation rate of the drug in Type III-B and Type-IV systems is generally higher than in Type-II and Type III-A systems [35]. The study conducted to compare the amount of precipitated APR in time in various formulations containing different amounts of APR is shown in Figure 9. It is clear that the concentration-time curves start from high concentration levels in formulations with a high cosolvent ratio, but also concentration decreases rapidly. That could be explained by if the cosolvents considerably contribute to drug solubilization, dilution of SNEDDS will cause the solvent capacity to decrease, leading the drug to precipitate [36].

As the cosolvent ratio decreases (<40%) and the surfactant ratio increases (>50%), the decline in the APR concentration slows down. Formulations with a high cosolvent content stabilized at a concentration of about 0.10 mg/mL after 4 h, while formulations with a lower content of cosolvents stabilized at a concentration of about 0.15 mg/mL. The mean areas under the concentration-time curves (AUC) are shown in Table 6.

It was determined that there was no correlation between the amount of APR that the formulations could dissolve and the AUC values. Although high loading capacity allowed the initial concentrations of the curve was high, the rapid decrease in concentration had a limiting effect on the AUC. It was observed that the highest AUC value was reached in formulations where the curve could remain constant concentration for a longer time. Thus, 20:60:20 formulation was picked for further development.

The precipitation test is a dissolution-like test but, unlike dissolution, it is performed under non-sink conditions. Distilled water [35] or various buffers [37] that mimic the gastric environment can be used as the medium. Since APR is a weakly basic drug, conditions are more challenging (in favor of precipitation) in distilled water than a gastric buffer. Thus, distilled water was selected as a dilution medium. The pH values of samples were measured between 3.5 and 4.5.

### 2.10. Selection of PPI for Super-SNEDDS

**Figure 10:**
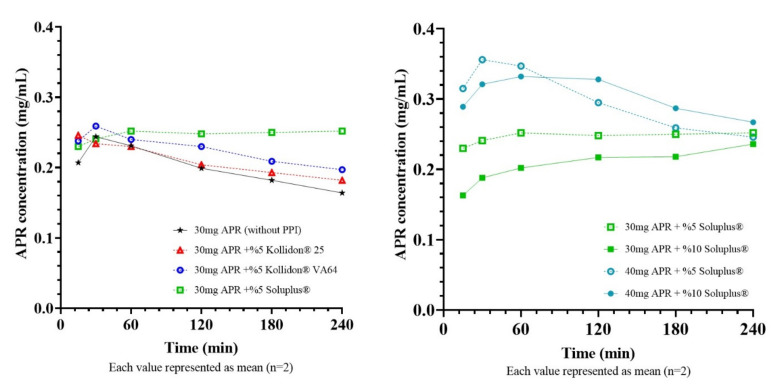
Concentration-time curves of super-SNEDDS formulations.

Equilibrium solubility of 20:60:20 formulation was found about 21 mg/mL. Super-SNEDDS were prepared by adding 30 mg (~150%) and 40 mg (~200%) to 1 g of blank 20:60:20 SNEDDS preconcentrate. It is vital to select a suitable PPI to maintain the drug in a supersaturated state for an elongated time while developing a supersaturated formulation. Since Methocel^TM^ E5 did not dissolve completely in formulations, only Kollidon^®^ 25, Kollidon^®^ VA64, Soluplus^®^ were tested as PPI. Concentration-time curves of diluted super-SNEDDS and corresponding AUC values were shown in Figure 10 and Table 7, respectively.

Super-SNEDDS is an attractive option since it prevents rapid drug precipitation and creates a supersaturated environment in the small intestine [38]. The logic behind the supersaturated formulation approach is that increased concentrations at the GI lumen can increase drug flux through the gastrointestinal epithelium, which can be explained by Fick’s first law [39]. In order to benefit from supersaturation in terms of bioavailability, this condition must be maintained for a sufficient time [40]. The test was continued for 240 min as the APR reaches its maximum plasma concentration in 240 min (Tmax). Results showed that Soluplus^®^ could delay the APR precipitation more effectively than other PPIs and provide a higher APR concentration for 240 min. Soluplus^®^ is a polymeric solubilizer with an amphiphilic chemical structure. It is capable of solubilizing poorly soluble drugs in aqueous media. Soluplus^®^ can form micelles of 70–100 nm in diameter above its critical micelle concentration (7.6 mg/L) [41]. Inhibition of drug precipitation can be accomplished by reducing the degree of supersaturation or delaying the drug precipitation [42]. The concentrations of Soluplus^®^ (0.5 g/L or 1g/L) in the test mediums were well above the critical micelle concentration. Thus, formed micelles can help to improve the drug solubility and inhibit the precipitation by reducing the degree of supersaturation.

The droplet size of nanoemulsion formed from super-SNEDDS was considerably higher than regular SNEDDS. Bannow et al. [43] also demonstrated that drug loads above the saturation solubility of simvastatin in the SNEDDS preconcentrate result in a significantly increased droplet size after emulsification. Additionally, PPI concentrations in preconcentrates had a notable influence on the droplet size. This result might indicate that at least a part of Soluplus^®^ incorporated into the emulsion droplets and cause droplets to grow.

### 2.11. In Vitro Lipolysis Test

20 mg APR loaded SNEDDS (~90% drug load) and 40 mg APR-loaded super-SNEDDS (~200% drug load) were tested in the lipolysis medium. Formulations were initially added to the vessels with an equal amount of APR. After the addition of pancreatic extract to the vessel, the digestion of the lipids was started. Since our tested formulations were Type III-B lipid-based drug delivery systems (LBDDS), the sample tubes had two distinct phases after centrifuge: an aqueous phase containing bile salts, fatty acids, mono-glycerides, and a pellet phase containing precipitated drug and calcium soap of fatty acids. Type II or III-A LBDDS can also have a third phase on the aqueous phase containing undigested triglycerides and diglycerides [44].

**Figure 11:**
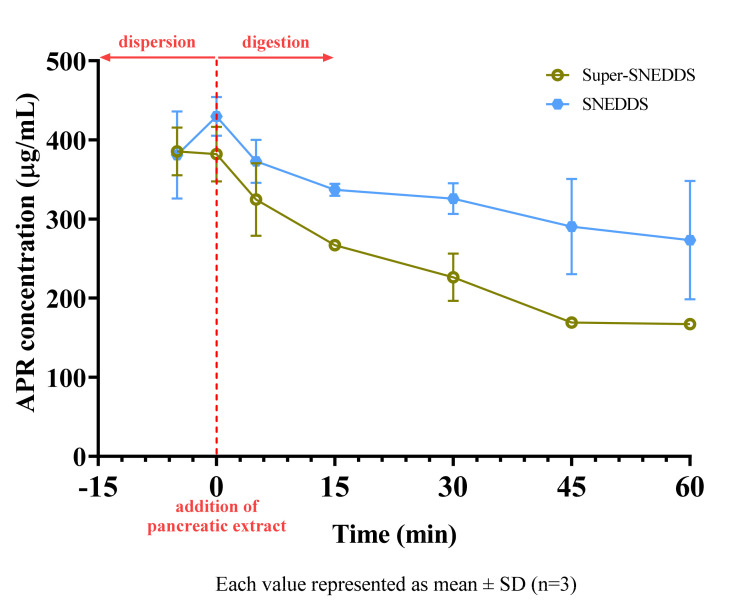
Concentration of APR during digestion of SNEDDS and super−SNEDDS.

The concentration of APR in the aqueous solution during the in vitro lipolysis test of SNEDDS and super-SNEDDS are shown in Figure 11. The solubilization capacity of both formulations decreased after start of digestion.

Arnold et al. studied digestion kinetics of various excipients. They reported that 93.6% of Imwitor^®^ 742, an excipient which is similar to Imwitor^®^ 988, and 34.7% of Kolliphor^®^ RH40 had been hydrolyzed after 3 h [45]. Since most of the formulations were composed of the mentioned excipients, we also observed the digestion of tested formulations. Hydrolysis of excipients caused a moderate decrease in the solubilization capacity of the drug. This effect was much more notable for super-SNEDDS formulation. Since the same mg of APR was added to vessels, SNEDDS vessels contained two-fold excipients. In other words, the excipient to drug ratio is higher for the SNEDDS than for the super-SNEDDS. Due to more excipients was remained unhydrolyzed (especially Kolliphor^®^ RH40) in SNEDDS vessels, more APR stayed dissolved. The results of in vitro lipolysis test showed that super-SNEDDS had no beneficial effect on the solubilization unless the applied volume of preconcentrates is a concern.

In vitro lipolysis test has been commonly employed for the in vitro evaluation of SNEDDS as it mimics in vivo conditions. However, the results of in vitro lipolysis test and bioavailability are not always compatible. Thomas et al. [34] demonstrated that if the drug precipitated in an amorphous form, the bioavailability of the drug might not be adversely affected. Increased drug load of super-SNEDDS preconcentrates makes them an attractive option, especially when rapid redissolution of amorphous precipitates does not limit bioavailability.

### 2.12. Preparation of Solid Super-SNEDDS and Selection of Carrier

Besides serious advantages, liquid SNEDDS has significant drawbacks such as incompatibilities of drugs with capsule material, low drug stability, drugs leakage, and capsule aging [46]. Solidified SNEDDS formulations could overcome these unwanted situations.

Firstly, maximum liquid super-SNEDDS formulation retention capacity for 1 g of each adsorbent was determined, then flowability of powders was investigated. The average weight of one drop of super-SNEDDS was approximately 14.5 mg. Maximum liquid retention capacity was found as 1.5 g (103 drops) for Syloid grades, 2 g (138 drops) for Neusilin grades, and 5 g (345 drops) for Florite^®^ R. Above these maximum amounts, powders were starting to become sticky and had a sludgy appearance. The flow properties of the adsorbents and solid super-SNEDDS are shown in Table 8. A total of 1 g liquid super-SNEDDS contain 35.09 mg APR. According to this data, APR equivalents of solid super-SNEDDS formulations are also presented in Table 8.

Neusilin^®^ US2 was selected as a porous carrier since it has a satisfying loading capacity with fair flowability. Loadig capacity of Neusilin^®^ US2 might be attributed to its large surface area. The flow property of a powder formulation is particularly important for the pharmaceutical industry. Hard capsule and sachet filling or tablet pressing might be challenging if the powder does not flow adequately. That will cause drug amounts in unit dosage forms to vary widely, which is unacceptable.

### 2.13. In Vitro Drug Release

**Figure 12:**
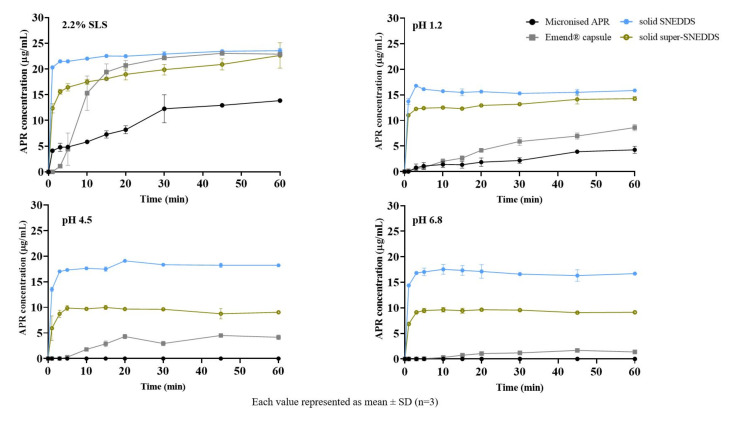
In vitro drug release from formulations in different mediums.

The in vitro drug release of solid SNEDDS (20 mg/g) and solid super-SNEDDS (40 mg/g) were performed in 2.2% SLS (FDA method) and various physiological buffers were shown in Figure 12. The release from solid SNEDDS was faster than super-SNEDDS. Soluplus^®^, a polymer used in super-SNEDDS to inhibit drug crystallization, could retain APR release since it was increasing the viscosity (visual observation) of the formulation. Increased viscosity will cause a prolongation of dispersion time. Additionally, solid SNEDDS released more APR than solid super-SNEDDS. That could be due to the higher amount of excipients in the solid SNEDDS formulation. The droplet size of the supersaturated formulation was significantly higher than the classical formulation, which leads to super-SNEDDS lesser surface area exposed to the dissolution medium.

APR release from the prepared formulations was considerably higher than micronized powder and marketed product, especially in physiological buffers. Faster drug release from the prepared formulations is attributed to the formation of nanoemulsions. The in vitro drug release from formulations prepared using adsorbents can be explained by physical interaction between adsorbent and medium is more powerful than the interaction between adsorbent and SNEDDS formulation [47].

### 2.14. Fourier Transform Infrared (FTIR) Spectroscopy

FTIR study was carried out to characterize possible interaction between drug and excipients. The spectrum of APR illustrated C=O stretching at 1700 cm^−1^, C-F stretching at 1130 cm^−1^, C-H stretching over the range 1500–1600 cm^−1^ [48]. All characteristic peaks according to the functional groups are present in its chemical structure.

**Figure 13:**
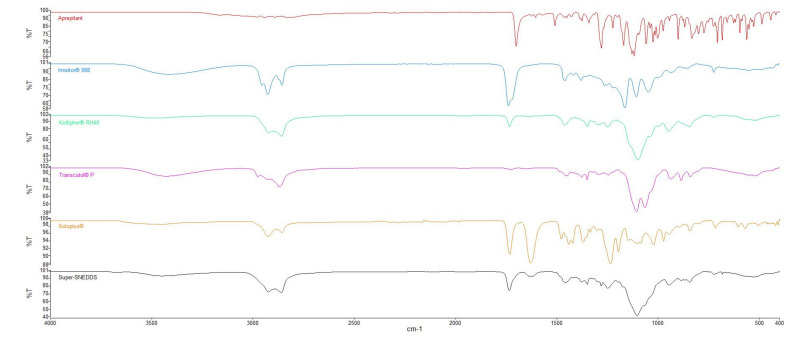
FTIR spectra of APR and excipients used in formulations.

Excipients that have hydroxyl groups such as Imwitor^®^ 988, Transcutol^®^ P, or Soluplus^®^ can form hydrogen bonds with APR. The C=O stretch peak of the APR shifted from 1700 cm^−1^ to 1734 cm^−1^. This shift could be attributed to hydrogen bonding between APR and the excipients used in the formulation. It was reported that Soluplus^®^ can form hydrogen bonds with drugs that consist of hydrogen bond acceptor groups such as C=O. Hydrogen bonding between polymers and drug molecules might lead to delayed nucleation and precipitation since nucleation activation energy was increased [49]. FTIR spectra of the APR and the excipients are shown in Figure 13.

## 3. Materials and Methods

### 3.1. Materials

APR was kindly gifted from Platin Kimya, Turkey. Imwitor^®^ 988 was kindly gifted from IOI Oleochem. Kolliphor^®^ RH40, Kolliphor^®^ ELP, Kolliphor^®^ HS15, Kolliphor^®^ P124, Soluplus^®^ were generously gifted from BASF (Ludwigshafen, Germany). Transcutol^®^ P, Capryol™ 90, Lauroglycol™ 90, Plurol^®^ Oleique CC, Lauroglycol™ FCC, Labrafil^®^ M 1944 CS, Labrafil^®^ M 2125 CS, Labrasol^®^ were generously gifted from Gattefossé (Lyon, France). Syloid^®^ XDP 3050 and 3150 were gifted from Grace (Columbia, MD, USA). Neusilin^®^ UFL2 and Neusilin^®^ US2 were gifted from Fuji chemical (Tokyo, Japan). Florite^®^ R was gifted from Tomita Pharmaceutical (Tokyo, Japan). HPLC grade methanol, Tween^®^ 20, Tween^®^ 60, Tween^®^ 80, and Brij^®^ O10 were purchased from Merck. Bile salt (B3883), pancreatic extract (P1625), 4-bromophenylboronic acid (B75956) were purchased from Sigma-Aldrich. Phospholipid (Lipoid S100) was purchased from Lipoid. All the other chemicals used were analytical grade.

### 3.2. Methods

#### 3.2.1. Method for Quantification of APR

HPLC (Thermo Surveyor, Temecula, CA, USA) equipped with a UV-vis detector was used for quantification of APR. Method validation was performed according to the ICH Q2 (R1) guideline. Waters Symmetry C18 (4.6 × 250 mm 5 µm) column was used for analyte separation. 80% (*v*/*v*) HPLC grade methanol and 20% (*v*/*v*) pH 3 aqueous phosphoric acid solution mixture was employed as mobile phase in isocratic elution mode with a flow rate of 0.8 mL/min. The column oven and sample tray temperature were set to 25 °C. The total run time of analysis was 10 min for the per run. A volume of 25 µL sample solution was injected and the APR was detected at a wavelength of 210 nm. APR standard solutions were prepared between 1–20 µg/mL concentrations to obtain the standard curve.

#### 3.2.2. Equilibrium Solubility of APR

The solubility of APR in various buffers, oils, surfactants, cosurfactants, cosolvents, and formulations was measured using the shake flask method. Briefly, an excess APR was added into a polypropylene tube that contains a medium. Tube mixed by a vortex (Daihan VM-10, South Korea) for 1 min to disperse APR. Mixtures were shaken at 100 rpm for 24 h at various temperatures in a thermostatically controlled shaking water bath (Nüve ST30, Turkey). Mixtures were then centrifuged at 13,500 rpm (12,225× *g*) for 15 min. The supernatant samples were suitably diluted with the mobile phase. Drug concentration was obtained via the validated HPLC method.

#### 3.2.3. Screening of Surfactants

The emulsification ability of various surfactants was screened by a method used by Date and Nagarsenker [33]. The turbidimetric method was used to evaluate the nanoemulsification efficiency of the surfactants. An equal amount of surfactant and selected oil were mixed. The mixture was heated at 60 °C and components were homogenized. 50 mg from the mixture was accurately weighed and then diluted with distilled water to 50 mL to yield fine nanoemulsion droplets. Emulsions were allowed to stand for 2 h then clarity was evaluated by determining the transmittance at 638.2 nm with UV-1601 (Shimadzu, Kyoto, Japan) spectrophotometer using distilled water as blank.

#### 3.2.4. Screening of Cosurfactants and Cosolvents

Screening of cosurfactants and cosolvents was performed using a similar turbidimetric method when surfactant screening. Oil, surfactant, and co-surfactant were weighed in the ratio of 3:2:1, respectively. The mixture was heated at 60 °C and components were homogenized. 50 mg from the mixture was accurately weighed and then diluted with distilled water to 50 mL to yield fine nanoemulsion droplets. Emulsions were allowed to stand for 2 h then clarity was evaluated by determining the transmittance at 638.2 nm with UV-1601 (Shimadzu, Japan) spectrophotometer using distilled water as blank. Since the oil ratio (50%) is equal to the oil ratio in the surfactant screening study, the transmittance value of resulting nanoemulsions will help to evaluate the relative nanoemulsification efficiency. The cosurfactant or cosolvent that increases the transmittance will increase the emulsification efficiency.

#### 3.2.5. Construction of Pseudo Ternary Phase Diagrams

The selected oil, surfactant, cosurfactant on the basis of solubility and excipient screening studies were used to develop the pseudo ternary phase diagrams using the water titration method. Various surfactant–cosurfactant (Smix) ratios were prepared using different proportions (1:3, 1:1, 3:1) of surfactant and cosurfactant. A series of oil/Smix mixtures were prepared at nine combinations (1:9, 2:8, 3:7, 4:6, 5:5, 6:4, 7:3, 8:2, and 9:1) and titrated with water to identify the nanoemulsion region. After each drop of water, the mixture was visually observed and transparent or turbid regions were noted. Pseudo ternary phase diagrams were drawn with the Tridraw 2.6 program. Calculation of the areas obtained from the pseudo ternary phase diagrams was performed with ImageJ 1.53e program.

#### 3.2.6. Preparation of SNEDDS Formulations

Blank SNEDDS preconcentrates were prepared by weighing the oil, surfactant, and cosurfactant in predetermined ratios and then homogenizing by heating at a mild (60 °C) temperature. SNEDDS was formed when the dispersion became transparent. Formulations containing the drugs were prepared by simply dissolving the APR in blank preconcentrates.

Saturated SNEDDS were prepared by adding excess APR into blank preconcentrates and then precipitate the insoluble part of APR. Briefly, SNEDDS and excess APR mixtures were shaken at 100 rpm for 24 h at 37 °C in a shaking water bath. Mixtures were then centrifuged at 13,500 rpm (12,225× *g*) for 15 min. The supernatant was separated carefully to obtain saturated SNEDDS.

Supersaturated SNEDDS formulations (super-SNEDDS) were prepared by adding 5% or 10% (*w*/*w*) various polymeric precipitation inhibitors (PPI) in blank SNEDDS preconcentrates. Following that, the desired amount of APR was added. The mixture was then mixed in a 60 °C water bath to facilitate solubilization. The heat was used in order to exceed the saturation concentration level. HPMC, PVP, PVP/VA, and Soluplus^®^ were examined as PPI.

#### 3.2.7. Nanoemulsion Droplet Size Measurements

The droplet size of the nanoemulsion was determined using a dynamic light scattering (DLS) particle size analyzer (Malvern Zetasizer Nano ZS, UK) which equipped a 633 nm laser. A total of 100 µL liquid SNEDDS was diluted 100-fold with distilled water before droplet size measurement. The droplet size of the resulting emulsion was determined by DLS at a scattering angle of 173° and 25 °C. All studies were repeated three times, and the values of Z-average diameters were used.

#### 3.2.8. In Vitro Drug Precipitation Test of Saturated SNEDDS

The precipitation of APR from the diluted formulation was determined as described by Pouton and Porter [35]. Saturated SNEDDS preconcentrates were diluted 100-fold with distilled water. The resulting nanoemulsions were kept in a shaking water bath at 37 °C and 100 rpm during the test. Further, 0.5 mL samples were taken without volume replacement at 15, 30, 60, 120, 180, and 240 min. The samples were filtered through a 0.45 μm nylon syringe filter, and the first half of filtrate was discarded. A total of 100 µL of the filtrate was taken and immediately diluted in 900 µL of mobile phase to prevent the precipitation. The concentration of APR in samples was measured by HPLC using the validated method. APR might exist either solubilized form (solubilized in medium or the emulsion droplets) or precipitated form after dilution of formulations with distilled water. Filtration of samples allows measuring the concentration of different forms of the drug except for the precipitated form [38].

The amount of dissolved APR until the 4 h were compared by calculating the area under the concentration-time curves (AUC), a method used by Quan et al. [49]. The AUC calculation was carried out according to the trapezoidal rule. SNEDDS were optimized by drug loading capacity, droplet stability, AUC of the concentration-time curve after dilution.

#### 3.2.9. Selection of PPI for Super-SNEDDS

Super-SNEDDS formulations were prepared as described in the “Preparation of SNEDDS formulations” section. Super-SNEDDS were diluted 100-fold with distilled water, and the test was carried out as explained in the “In vitro drug precipitation test from saturated SNEDDS” section. Kollidon^®^ 25 (PVP), Kollidon^®^ VA64 (PVP/VA), Methocel^TM^ E5 (HPMC), Soluplus^®^ were evaluated as PPI. Experiments were carried out by adding 5% and 10% (*w*/*w*) of the mentioned polymers to the prepared formulations. The drug concentration-time profiles of super-SNEDDS were determined. The effect of added PPIs on APR precipitation was investigated.

#### 3.2.10. In Vitro Lipolysis Test

Lipolysis medium (pH 6.5) for in vitro digestion experiments were as follows: 2 mM Tris-maleate, 1.4 mM CaCl_2_·2H_2_O, 50 mM NaCl, 2.95 mM bile salt and 0.26 mM phospholipid. The lipolysis medium and formulations were added to temperature-controlled (37 °C) vessel. The 15 min dispersion step was employed to facilitate complete dispersion of the formulations before the start of digestion. Pancreatin from porcine pancreas containing pancreatic lipase was prepared by mixing 1 g of pancreatin powder in 5 mL digestion buffer (free of bile salt and phospholipid) for 15 min. Then pH of the mixture was adjusted to 6.5. The enzyme suspension was centrifuged at 4000 rpm (2075× *g*). The pancreatic extract obtained was added to the lipolysis medium to initiate digestion, after 15 min of dispersion. To minimize loss of enzyme activity pancreatin extract was prepared freshly using cold digestion buffer before each test. The pH during the test was manually adjusted to pH 6.5 ± 0.05 using small quantities of 0.4 M NaOH. Samples were withdrawn after 10 and 15 min of dispersion and after 5, 15, 30, 45, and 60 min of digestion. The lipase activity was inhibited immediately by the addition of 5 μL 4-BBBA into the samples. Samples were centrifuged at 13,500 rpm (12,225× *g*) for 15 min. APR in the supernatant was measured by HPLC using the validated method after appropriate dilution.

#### 3.2.11. Preparation of Solid Super-SNEDDS and Selection of Carrier 

The solid super-SNEDDS formulations were prepared by adsorbing super-SNEDDS formulation on porous carriers. Five different adsorbents were used: Syloid^®^ XDP 3050 and Syloid^®^ XDP 3150, Neusilin^®^ UFL2, Neusilin^®^ US2, and Florite^®^ R. In addition, 1 g of each adsorbent was placed separately in a mortar and the formulations were added dropwise. The mixture was mixed with a pestle after each addition. Maximum liquid retention ability for each adsorbent was determined.

The flow properties of the solid super-SNEDDS were confirmed by bulk density, tapped density, Carr’s compressibility index, and Hausner ratio. The bulk density of powder was found simply by dividing the mass of the powder by its volume. The tapped density was measured using a tap density analyzer (Pharmatest, Hainburg, Germany). A total of 500 and 1250 taps were carried out on the same powder sample in a graduated cylinder. If the powder volume decreased, the sample was tapped 1250 times until it was unchanged. The unchanged final volume was the tapped volume. The tapped density was calculated by dividing powder mass by tapped volume.

#### 3.2.12. In Vitro Drug Release

In vitro drug release test was conducted by using a dissolution tester (Pharmatest, Hainburg, Germany). Dissolution studies of APR-loaded solid SNEDDS were carried out using the USP paddle method (Apparatus II) at 100 rpm and 37 °C. An amount of 900 mL of 2.2% sodium lauryl sulfate in distilled water, pH 1.2 HCl buffer, pH 4.5 acetate buffer, and pH 6.8 phosphate buffer were used as the dissolution mediums. Further, 1 mL samples from dissolution vessels were withdrawn at 1,3, 5, 10, 15, 20, 30, 45, and 60 min and filtered through a 0.45 µm syringe filter. The dissolution medium was made up to 900 mL with a fresh medium to maintain a constant volume. APR concentrations were determined by the validated HPLC method without any dilution. Dissolution tests of prepared formulations, marketed capsules, and APR powder were performed for comparison. Each dissolution vessel contained the equivalent of 20 mg of APR.

#### 3.2.13. Fourier Transform Infrared (FTIR) Spectroscopy

FTIR measurements were carried out using an infrared spectrophotometer (Perkin Elmer Spectrum Two, USA) in attenuated total reflectance mode fitted with a ZnSe crystal. The scanning range was 400–4000 cm^−1^, and the resolution was 2 cm^−1^. The spectra of the APR and various formulations were recorded. 

## 4. Conclusions

In the present research, SNEDDS formulations of APR were successfully prepared with different approaches. Besides classical SNEDDS, super-saturated and solid formulations were also prepared and characterized. Preliminary studies and pseudo ternary phase diagram analysis suggested that formulation contains 20% Imwitor^®^ 988, 60% Kolliphor^®^ RH40, and 20% Transcutol^®^ P by weight showed appropriate properties. Soluplus^®^ was used as a PPI, and Neusilin^®^ US2 was used as a porous carrier in addition to classic SNEDDS formulation. Formulations characterized by DLS, FTIR, in vitro lipolysis test, and dissolution test. The study showed that the lipid-based drug delivery technique was a successful and simple approach to prepare a new APR solid dosage form and to enhance its dissolution rate. Overall, the developed SNEDDS formulations with increased solubility of APR could be a better alternative to micronized APR or marketed capsules.

## Figures and Tables

**Table 1:** Results of surfactant screening for Imwitor^®^ 988.

Surfactant	% Transmittance	Appearance
Kolliphor^®^ RH40	98.56	Clear
Kolliphor^®^ ELP	97.47	Clear
Kolliphor^®^ HS15	96.00	Clear
Tween^®^ 20	87.83	Bluish
Tween^®^ 60	84.78	Bluish
Tween^®^ 80	75.27	Bluish white
Kolliphor^®^ P124	45.80	Grayish white
Brij^®^ O10	45.15	Grayish white
Labrafil^®^ M 2125 CS	19.25	Grayish white
Labrasol^®^	16.92	Grayish white
Labrafil^®^ M 1944 CS	11.18	Grayish white

**Table 2:** Results of cosurfactant and cosolvent screening for Imwitor^®^ 988.

Oil	Surfactant	Cosurfactant or Cosolvent	% Transmittance	Appearance
Imwitor^®^ 988	Kolliphor^®^ RH40	Capryol™ 90	99.68	Clear
		PEG 200	99.62	Clear
		Lauroglycol™ 90	99.33	Clear
		Transcutol^®^ P	99.28	Clear
		Propylene glycol	98.91	Clear
		PEG 400	98.90	Clear
		Plurol^®^ Oleique CC	98.88	Clear
		* Kolliphor^®^ RH40	98.56	Clear
		Lauroglycol™ FCC	97.33	Clear
	Kolliphor^®^ ELP	PEG 200	99.77	Clear
		PEG 400	99.62	Clear
		Capryol™ 90	99.48	Clear
		Lauroglycol™ FCC	99.08	Clear
		Propylene glycol	99.05	Clear
		* Kolliphor^®^ ELP	97.47	Clear
		Transcutol^®^ P	97.30	Clear
		Lauroglycol™ 90	95.58	Clear
		Plurol^®^ Oleique CC	70.13	Bluish white
	Kolliphor^®^ HS15	PEG 200	99.90	Clear
		Transcutol^®^ P	99.74	Clear
		PEG 400	99.55	Clear
		Propylene glycol	99.46	Clear
		Capryol™ 90	98.93	Clear
		Lauroglycol™ FCC	97.51	Clear
		* Kolliphor^®^ HS15	96.00	Clear
		Lauroglycol™ 90	85.46	Bluish
		Plurol^®^ Oleique CC	75.66	Bluish white

* Shaded rows indicate the results of surfactant screening (50% oil–50% surfactant)

**Table 3:** Clear nanoemulsion areas of the pseudo ternary phase diagrams (as unit^2^).

Oil	Surfactant	Cosolvent	S_mix_ (Surfactant: Cosolvent)			Total Area
			1:1	3:1	1:3	
Imwitor^®^ 988	Kolliphor^®^ RH40	Transcutol^®^ P	335.1	352.5	343.8	1031.4
	Kolliphor^®^ ELP		337.2	307.3	328.4	972.9
	Kolliphor^®^ HS15		321.8	280.8	320.2	922.8

**Table 4:** Z-average and PDI values of blank formulations up to 30% Imwitor^®^ 988.

Formulation Code	Z-Average (nm)	PDI
10:10:80	30.27 ± 8.55	0.319
10:20:70	20.44 ± 5.65	0.305
10:30:60	15.02 ± 2.43	0.105
10:40:50	15.05 ± 2.63	0.122
10:50:40	13.81 ± 1.98	0.083
10:60:30	13.36 ± 1.48	0.049
10:70:20	14.22 ± 2.28	0.102
10:80:10	14.57 ± 2.23	0.094
20:10:70	30.44 ± 4.35	0.082
20:20:60	30.60 ± 9.08	0.352
20:30:50	22.45 ± 6.40	0.325
20:40:40	16.12 ± 2.25	0.078
20:50:30	15.38 ± 2.32	0.091
20:60:20	14.41 ± 2.07	0.083
20:70:10	14.47 ± 2.55	0.124
30:10:60	38.66 ± 4.36	0.051
30:20:50	27.44 ± 5.01	0.133
30:30:40	45.31 ± 15.98	0.498
30:40:30	48.62 ± 17.93	0.543
30:50:20	24.09 ± 7.48	0.386
30:60:10	16.42 ± 2.56	0.097

Each value represented as mean ± SD (*n* = 3).

**Table 5:** Stability of droplets after dilution with distilled water.

Loaded APR		21 mg		25 mg		30 mg	
% Ratio (O:S:Cs)	Time	Z-Average (nm)	PDI	Z-Average (nm)	PDI	Z-Average (nm)	PDI
30:10:60	0 h	48.06 ± 5.10	0.045	49.77 ± 3.73	0.022	52.24 ± 5.56	0.045
	1 h	48.20 ± 5.53	0.053	49.52 ± 6.78	0.075	52.97 ± 8.69	0.108
	2 h	47.52 ± 25.04	0.035	53.99 ± 24.73	0.266	96.28 ± 24.67	0.288
	4 h	48.97 ± 8.53	0.121	124.8 ± 28.22	0.204	130.8 ± 33.3	0.260
20:10:70	0 h	36.69 ± 2.19	0.014	38.21 ± 4.16	0.047	38.96 ± 4.24	0.047
	1 h	38.40 ± 5.34	0.077	38.97 ± 5.32	0.074	39.84 ± 7.25	0.133
	2 h	36.87 ± 4.91	0.071	44.60 ± 9.35	0.176	63.63 ± 12.88	0.164
	4 h	161.2 ± 45.02	0.312	546.5 ± 214.2	0.614	674.1 ± 322.0	0.913
30:20:50	0 h	28.08 ± 2.08	0.022	29.19 ± 3.79	0.068	28.96 ± 3.23	0.050
	1 h	28.18 ± 1.63	0.013	28.17 ± 2.14	0.023	28.77 ± 2.28	0.025
	2 h	28.03 ± 3.22	0.053	27.95 ± 3.43	0.060	30.77 ± 8.01	0.271
	4 h	29.14 ± 6.75	0.215	118.4 ± 26.10	0.194	3059 ± 1530	1
10:30:60	0 h	23.16 ± 5.96	0.265	20.07 ± 5.48	0.298	496.7 ± 248.4	1
	1 h	16.55 ± 2.73	0.109	2686 ± 1343	1	649.6 ± 275.6	0.720
	2 h	205.3 ± 57.75	0.317	1054 ± 508	0.927	4637 ± 2319	1
	4 h	1897 ± 949	1	2568 ± 1284	1	4673 ± 2337	1
10:40:50	0 h	14.40 ± 2.23	0.096	15.36 ± 3.02	0.154	14.27 ± 1.43	0.040
	1 h	14.54 ± 1.98	0.083	16.29 ± 3.76	0.213	75.08 ± 14.99	0.160
	2 h	14.60 ± 1.86	0.062	26.51 ± 5.57	0.177	728.9 ± 327.3	0.806
	4 h	14.66 ± 1.70	0.048	194.8 ± 59.85	0.377	1118 ± 559	0.969
20:40:40	0 h	21.25 ± 5.84	0.302	24.33 ± 7.49	0.379	not dissolved	
	1 h	20.36 ± 5.24	0.265	22.76 ± 6.75	0.352		
	2 h	22.13 ± 6.29	0.323	22.68 ± 6.99	0.380		
	4 h	103.0 ± 19.14	0.138	1828 ± 898	0.964		
10:50:40	0 h	13.99 ± 2.38	0.116	13.54 ± 1.22	0.033	not dissolved	
	1 h	14.01 ± 3.66	0.123	14.56 ± 2.65	0.133		
	2 h	15.03 ± 1.07	0.141	445 ± 178.4	0.643		
	4 h	15.26 ± 3.00	0.154	856.6 ± 400.9	0.876		
20:50:30	0 h	15.46 ± 2.26	0.086	not dissolved		not dissolved	
	1 h	15.52 ± 2.05	0.071				
	2 h	15.60 ± 1.90	0.062				
	4 h	15.58 ± 1.86	0.057				
10:60:30	0 h	13.67 ± 1.82	0.071	not dissolved		not dissolved	
	1 h	13.71 ± 1.61	0.055				
	2 h	13.74 ± 1.47	0.046				
	4 h	13.80 ± 1.59	0.053				
20:60:20	0 h	14.48 ± 1.80	0.061	not dissolved		not dissolved	
	1 h	14.78 ± 1.84	0.065				
	2 h	14.85 ± 1.89	0.068				
	4 h	15.14 ± 2.03	0.072				

Each value represented as mean ± SD (*n* = 3).

**Table 6:** AUC of saturated SNEDDS formulations after dilution with distilled water.

Formulation	AUC (mg·min·mL^−1^)
30:10:60	31.380
20:10:70	23.888
10:30:60	28.478
30:20:50	24.473
10:40:50	26.438
20:40:40	26.850
10:50:40	29.753
20:50:30	35.910
10:60:30	36.458
20:60:20	38.558
10:70:20	36.353
30:60:10	32.205
10:80:10	30.728
20:70:10	29.258

Each value represented as mean (*n* = 2).

**Table 7:** AUC of super-SNEDDS formulations after dilution with distilled water.

Formulation	AUC (mg.min.mL^−1^)
30 mg APR (without PPI)	45.206
30 mg APR + 5% Kollidon^®^ 25	46.705
30 mg APR + 5% Kollidon^®^ VA64	50.702
30 mg APR + 5% Soluplus^®^	55.916
30 mg APR + 10% Soluplus^®^	47.676
40 mg APR + 5% Soluplus^®^	66.621
40 mg APR + 10% Soluplus^®^	69.270

Each value represented as mean (*n* = 2).

**Table 8:** Flow properties of adsorbents and various solid super-SNEDDS.

Powder	Bulk Density (g/mL)	Tapped Density (g/mL)	Hausner Ratio	Carr’s Index	Flowability
Syloid^®^ XDP 3050	0.233	0.286	1.23	18.53	Fair
Syloid^®^ XDP 3150	0.244	0.278	1.14	12.23	Good
Neusilin^®^ UFL2	0.104	0.179	1.72	41.90	Very, very poor
Neusilin^®^ US2	0.179	0.213	1.19	15.96	Fair
Florite^®^ R	0.077	0.127	1.65	39.37	Very, very poor
super-SNEDDS: Neusilin^®^ UFL2 2 g (70.2 mg APR): 1 g	0.359	0.511	1.42	29.75	Poor
super-SNEDDS: Neusilin^®^ US2 2 g (70.2 mg APR): 1 g	0.409	0.573	1.40	28.62	Poor
super-SNEDDS: Neusilin^®^ US2 1.5 (52.6 mg APR): 1 g	0.390	0.476	1.22	18.07	Fair
super-SNEDDS: Florite^®^ R 5 g (175.5 mg APR): 1 g	0.243	0.532	2.19	54.32	Very, very poor
super-SNEDDS: Florite^®^ R 3 g (105.3 mg APR): 1 g	0.298	0.474	1.59	37.13	Very poor

## Data Availability

Data is contained within the article.
